# miR-221/222 Compensates for Skp2-Mediated p27 Degradation and Is a Primary Target of Cell Cycle Regulation by Prostacyclin and cAMP

**DOI:** 10.1371/journal.pone.0056140

**Published:** 2013-02-07

**Authors:** Paola Castagnino, Devashish Kothapalli, Elizabeth A. Hawthorne, Shu-Lin Liu, Tina Xu, Shilpa Rao, Yuval Yung, Richard K. Assoian

**Affiliations:** 1 Department of Pharmacology, Perelman School of Medicine, University of Pennsylvania, Philadelphia, Pennsylvania, United States of America; 2 Molecular Profiling Facility, Perelman School of Medicine, University of Pennsylvania, Philadelphia, Pennsylvania, United States of America; University Magna Graecia, Italy

## Abstract

p27^kip1^ (p27) is a cdk-inhibitory protein with an important role in the proliferation of many cell types. SCF^Skp2^ is the best studied regulator of p27 levels, but Skp2-mediated p27 degradation is not essential in vivo or in vitro. The molecular pathway that compensates for loss of Skp2-mediated p27 degradation has remained elusive. Here, we combine vascular injury in the mouse with genome-wide profiling to search for regulators of p27 during cell cycling in vivo. This approach, confirmed by RT-qPCR and mechanistic analysis in primary cells, identified miR-221/222 as a compensatory regulator of p27. The expression of miR221/222 is sensitive to proteasome inhibition with MG132 suggesting a link between p27 regulation by miRs and the proteasome. We then examined the roles of miR-221/222 and Skp2 in cell cycle inhibition by prostacyclin (PGI_2_), a potent cell cycle inhibitor acting through p27. PGI_2_ inhibited both Skp2 and miR221/222 expression, but epistasis, ectopic expression, and time course experiments showed that miR-221/222, rather than Skp2, was the primary target of PGI_2_. PGI_2_ activates Gs to increase cAMP, and increasing intracellular cAMP phenocopies the effect of PGI_2_ on p27, miR-221/222, and mitogenesis. We conclude that miR-221/222 compensates for loss of Skp2-mediated p27 degradation during cell cycling, contributes to proteasome-dependent G1 phase regulation of p27, and accounts for the anti-mitogenic effect of cAMP during growth inhibition.

## Introduction

p27^kip1^ (p27), an inhibitor of cyclin-dependent kinases (cdks), has a critical regulatory role in the proliferation of many cell types. p27 inhibits the activation of cyclin-cdk2 and cyclin-cdk1 complexes, with cyclin E-cdk2 being the major p27 target in G1 phase. p27 (along with its family members, p21^cip1^ and p57^kip1^) also has a pro-proliferative effect as an assembly factor for cyclin D-cdk4/6 complexes in G1 phase [1], but the *hyper*proliferative phenotype seen upon deletion of p27 in mice [2] indicates that the inhibitory effect of p27 on cdk activity is usually dominant in vivo. In agreement with this notion, p27 levels are high in quiescent cells and downregulated in mid-late G1 phase in response to soluble mitogens. Mechanisms regulating the expression, localization, and degradation of p27 have been intensely examined [3].

The best studied mechanism for regulating p27 levels involves proteosomal degradation by SCF^Skp2^ [4]. Skp2 is the substrate-specificity component within this complex; it recognizes p27 that has been phosphorylated by cyclin E-cdk2 on T187 and targets p27 for ubiquitinylation and ubiquitin-mediated proteolysis [5], [6]. However, mice harboring a Skp2-resistant p27 allele (p27T187A) show near normal p27 regulation and proliferative capacity [7]. Similarly, cells isolated from p27T187A mice display normal downregulation of p27 in G1 phase although S phase expression of p27 is disrupted by the T187A mutation [7]. This surprising finding has led to the notion that there must be mechanisms compensating for loss of Skp2-mediated p27 degradation. In addition to Skp2-dependent proteolysis, previous studies have identified T187A-independent proteosomal degradation, perhaps through Skp2, KPC, or Cul4A and 4B [7]–[9]. In addition, p27 levels can be regulated transcriptionally, by changes in protein translation, and by caspase-mediated degradation [10]–[14]. Most recently, miR-221/222 has emerged as a novel regulator of p27 [15]–[18]. miR-221/222 is a bicistronic microRNA family that directly suppresses p27 levels post-transcriptionally by binding to two discrete sites in the p27 mRNA 3′UTR [15]–[17]. Which if any of these molecules and pathways compensate for the loss of Skp2-mediated degradation remains unknown.

p27 has a particularly important role in the proliferation of vascular smooth muscle cells (VSMCs). Loss of p27 accelerates atherosclerosis in apoE-null mice [19] whereas upregulated expression of p27 contributes to the anti-proliferative effect of rapamycin after vascular injury [20], [21]. We and others have reported that stable mimetics of the anti-mitogen, prostacyclin (PGI_2_), increase the levels of p27 in cultured VSMCs and in vivo [22]–[24]. This effect on p27 is causally related to the anti-mitogenic action of PGI_2_ because PGI_2_ mimetics fail to inhibit S phase entry in p27-null VSMCs [22]. PGI_2_ mimetics also inhibit the mitogen-dependent induction of Skp2 [24], but the finding that Skp2 is dispensable for p27 regulation means that the simple model of PGI_2_ acting on p27 through Skp2 must be incomplete. In this report, we combined vascular injury in the mouse with transcript profiling to identify miR-221/222 as the compensatory regulator of p27. Additionally, we show that miR-221/222 plays a primary role in cell cycle inhibition by PGI_2_ and its canonical second messenger, cAMP.

## Results

### Skp2-mediated p27 Degradation is Dispensable for VSMC Cycling in vivo

We examined the relative effects of p27 and Skp2 on VSMC proliferation in vivo by comparing the response to vascular injury in WT, p27-null and p27T187A mice. In this model, a fine wire is passed through the lumen of a femoral artery to denude the intimal endothelial layer. Platelets aggregate and degranulate at these sites of endothelial damage, and this effect leads to the dedifferentiation, migration and proliferation of VSMCs in the underlying medial layer. VSMC migration and proliferation ultimately results in the narrowing of the arterial lumen, a response that can be quantified morphometrically by calculating the percentage luminal stenosis (percent of the lumen that is occluded).

The uninjured arteries of wild-type, p27-null or T187A-null mice were not grossly distinguishable (Fig. S1A). Deletion of p27 increased the response to injury (Fig. S1A–B) as compared to wild-type mice, consistent with the established role of p27 as a cdk inhibitor. However, knock-in of p27T187A did not suppress the response to vascular injury (Fig. S1A–B). Ki67 staining confirmed that in vivo VSMC cycling was increased by deletion of p27 but not reduced, relative to wild-type, when Skp2-dependent degradation of p27 was blocked by the T187A mutation (Fig. S1C–D). Thus, Skp2-mediated p27 degradation is dispensable for VSMC cycling in vivo. We noted a significant difference in Ki67 labeling between p27-null and p27T187A mice that was not detected in luminal stenosis between the same lines (compare Figs. S1B and S1D). This difference may reflect the reported stimulatory effect of cytoplasmic p27 on cell migration [25]. Both migration and proliferation contribute to the degree of luminal stenosis, but only proliferation would be scored by Ki67 labeling. The relative effects of p27 deletion and T187A knock-in on VSMC proliferation and migration likely differ, and this confounds direct comparisons between percent stenosis and cell cycling in injured p27-null and p27T187A mice.

### miR-221/222 Compensates for Loss of Skp2-mediated p27 Degradation

**Figure 1:**
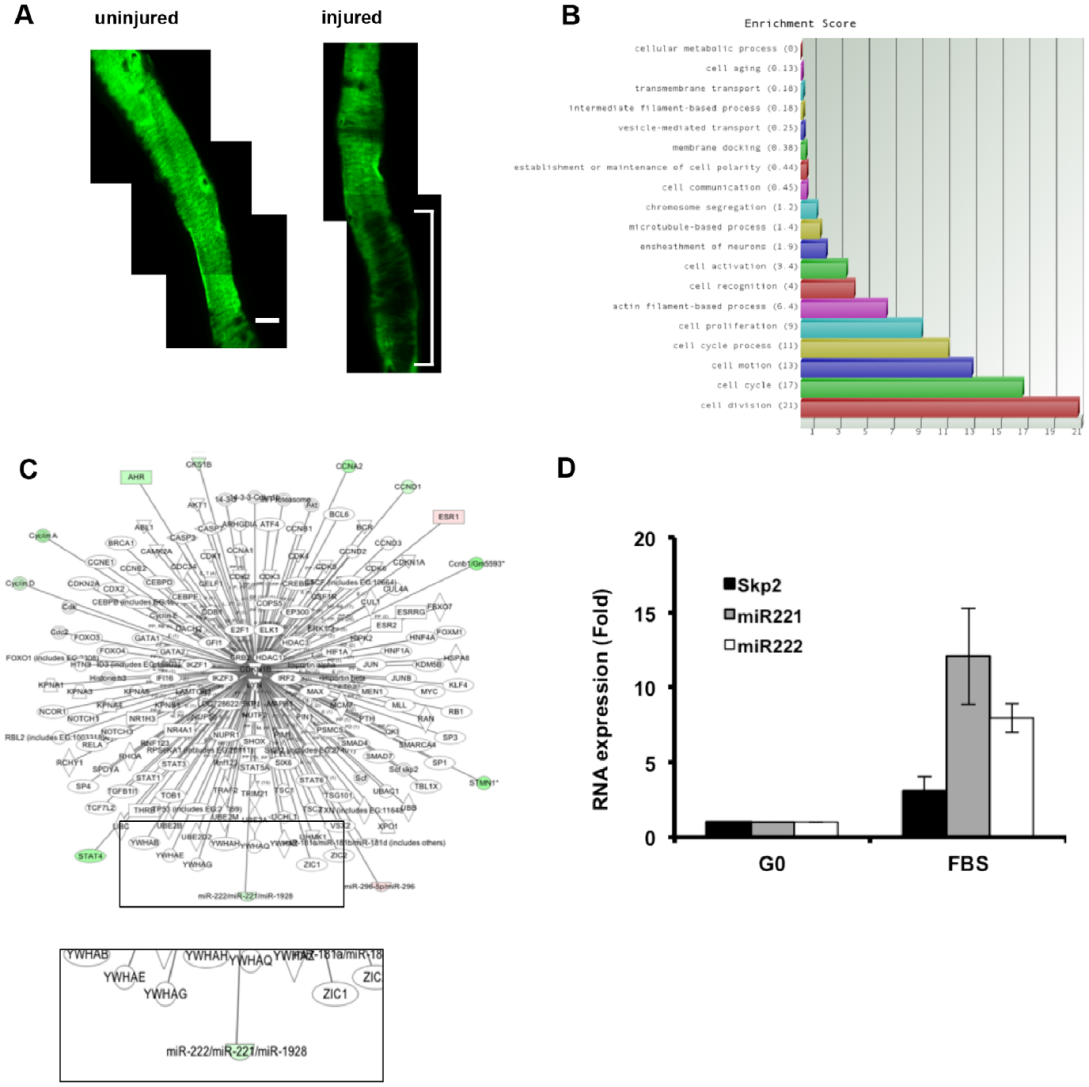
Transcript profiling reveals that miR-221/222 is induced after vascular injury in vivo. (**A**) Male SMA-GFP mice (5–6 mo) were subjected to fine-wire femoral artery injury. Injured arteries were isolated, carefully opened, and immediately imaged for GFP fluorescence. A representative image of an uninjured and injured femoral artery is shown for a single mouse. The bracket shows a region of vascular injury. (**B**) Uninjured femoral arteries and GFP-negative regions of injured femoral arteries were collected for transcript profiling. Genes differentially expressed in these tissues were plotted against the Gene Ontology (GO) category, Cellular Process. (**C**) Interaction map showing upstream regulators of p27 that are differentially expressed in injured vs. uninjured femoral arteries as determined by Ingenuity Pathway Analysis (IPA) of the microarray data. Green and red represent induction and repression, respectively. Upstream p27 regulators in the IPA database that were not differentially expressed during in vivo response to injury are uncolored. The boxed region of interest at the bottom of the interaction map is expanded below to highlight the induction of miR-221 (green oval). (**D**) Quiescent early passage mouse VSMCs were stimulated with 10% FBS for 24 h. Total RNA was collected, and the levels of miR-221/222 and Skp2 mRNA were determined by RT-qPCR. Results show mean ± SD, n = 2.

We injured the femoral arteries of SMA-GFP transgenic mice, a line in which the α-smooth muscle actin promoter drives GFP [26]. Since the SMA promoter is expressed in differentiated non-cycling VSMCs, vascular injury in this mouse line reports VSMC dedifferentiation and proliferation by the loss of GFP fluorescence (Fig. 1A; bracket), a conclusion supported by direct analysis of BrdU-labeled nuclei [27]. We microdissected these regions of cycling VSMCs and determined global mRNA and miRNA expression relative to uninjured, non-cycling contralateral controls. Differentially expressed mRNAs were identified using statistical analysis of microarrays algorithm and subjected to enrichment analysis against the Gene Ontology (GO) database [28]; the GO categories associated with cell proliferation and cycling scored among the highest differentially expressed genes (Fig. 1B and Table S1). Differentially expressed miRNAs were identified similarly, and the list of differentially expressed miRNAs (Table S2) and cell cycle mRNAs were superimposed on the Ingenuity database of all molecules that have been experimentally demonstrated to target p27 directly. This analysis identified miR-221 as a differentially expressed upstream regulator of p27 (Fig. 1C). Differential expression of other p27 regulators, including the genes for FOXO transcription factors, Cul4A, Cul4B, and Skp2 was not detected. Directed analysis by RT-qPCR confirmed the induction of miR-221 and also revealed induction of its bicistronic partner, miR-222, in the injured arteries of 5 of 6 additional SMA-GFP mice (Table 1). Skp2 mRNA was poorly induced in these samples (Table 1). miR-221/222 induction was also seen after FBS stimulation of primary VSMCs in vitro (Fig. 1D), and in agreement with our in vivo data, the magnitude of the FBS effect on miR-221/222 exceeded that seen for Skp2 mRNA (Fig. 1D).

**Table 1:** miR-221/222 and Skp2 mRNA expression in injured femoral arteries.

Fold increase (injured/uninjured)			
*Mouse #*	miRNA221	miRNA222	Skp2
**101**	0.3	0.2	ND
**103**	7.1	6	ND
**104**	10	8	ND
**131**	6	5	2
**132**	38	48	2
**272**	5	6.8	1.8

Male SMA-GFP (5–6 mo.) mice were subjected to fine-wire femoral artery injury. Injured regions of femoral arteries (as shown in Fig. 1A) and uninjured control femoral arteries were micro-dissected, and RNA was isolated. RT-qPCR was performed for miRNA221, miRNA222 and Skp2 expression. Gene expression for each mouse was expressed as fold increase (injured regions relative to uninjured control).

**Figure 2:**
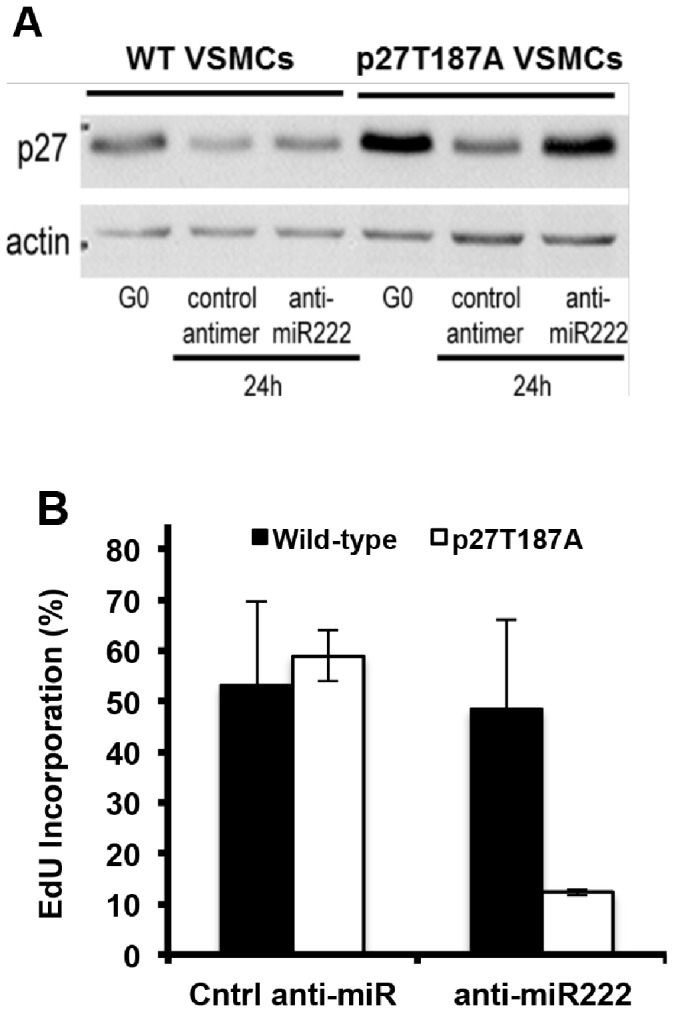
Effects of miR-221/222 and Skp2 on p27 levels during cell cycling and cell cycle inhibition. (**A**) Early passage VSMCs from wild-type or p27T187A mice were transiently transfected with control or anti-miR-222. Transfected cells were serum starved and stimulated with 10% FBS for 24 h before being collected and analyzed by western blotting for p27 and actin (loading control). (**B**) Cells were also serum starved and stimulated with 10% FBS for 48 h on cover slips; collected coverslips were used to determine EdU incorporation. Results show mean ± SD, n = 3.

Skp2 levels can be controlled post-transcriptionally as well as transcriptionally [29]–[31], so the lack of Skp2 mRNA induction in vivo does not preclude a role for Skp2 protein in p27 regulation. To directly compare the roles of miR-221/222 and Skp2 on p27 regulation, we transfected wild-type and p27T187A VSMCs with a modified RNA oligonucleotide complementary to miR-222 (hereafter called the “anti-miR”) that effectively inhibits the expression of both miR-221 and 222 ([32] and Fig. S2). Serum-stimulated downregulation of p27 (Fig. 2A) and incorporation of EdU (Fig. 2B) were only slightly reduced in wild-type VSMCs expressing anti-miR-222 or in cells expressing the p27T187A allele. In contrast, miR-221/222 inhibition in p27T187A cells efficiently blocked the FBS-induced downregulation of p27 and S phase entry (Fig. 2A–B). Thus, miR-221/222 and Skp2 redundantly contribute to the down-regulation of p27.

### Regulation of miR221/222 and Skp2 by PGI_2_

**Figure 3:**
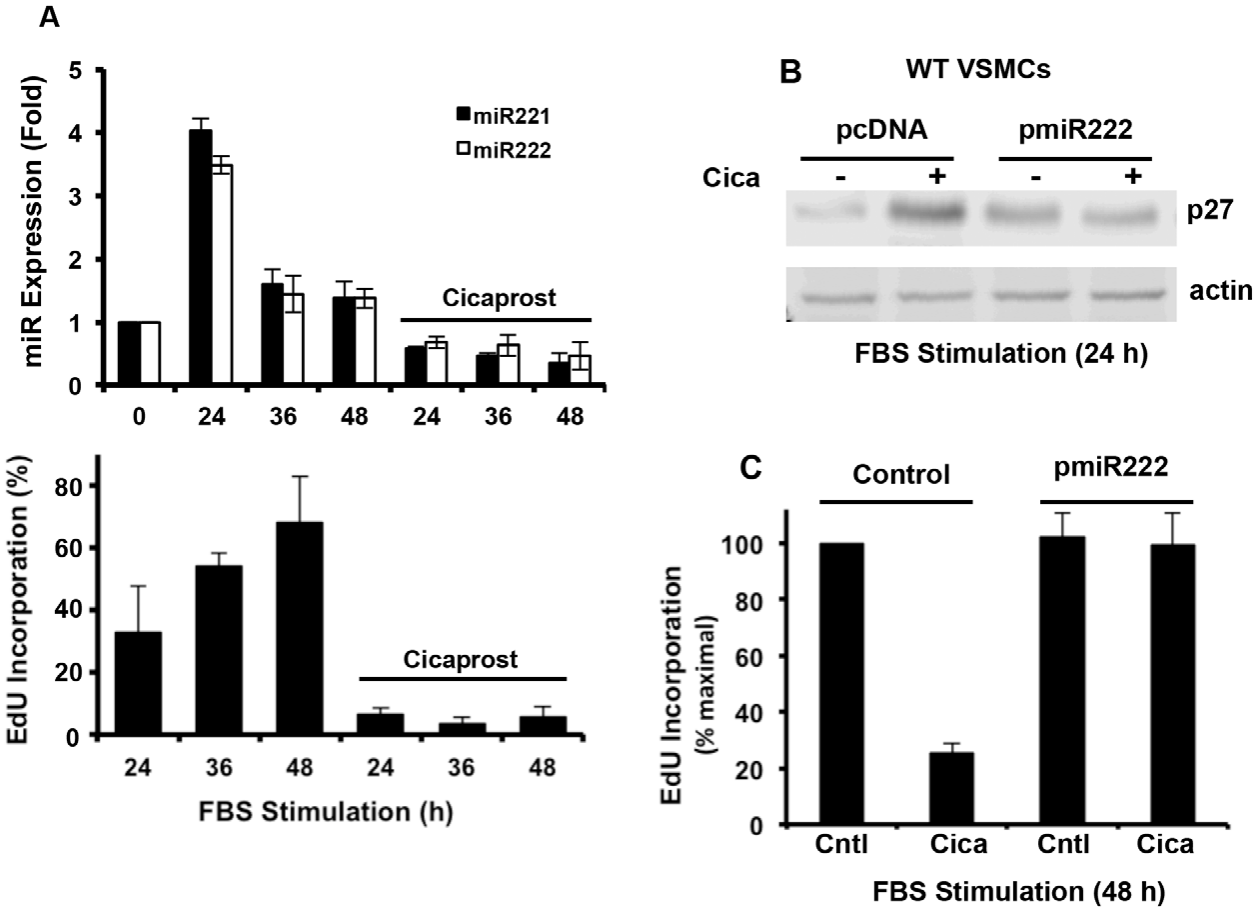
miR221/222 regulation by PGI2 and role in mitogensis. (**A**) Serum-starved VMSCs from wild-type mice were incubated with 10% FBS and 2 nM cicaprost for selected times in the presence of EdU. Top Results show mean ± SD, n = 2. Bottom panel: S phase entry was assessed and plotted as percent maximal EdU incorporation. Results show mean ± SD, n = 3. (**B–C**) Early passage wild-type VSMCs were transiently transfected with an expression plasmid for pCDNA (control) or miRNA-222. The cells were serum starved and stimulated with 10% FBS for 24 (panel B) or 48 (panel C) h in the absence or presence of 200 nM cicaprost (cica) before being collected and analyzed for p27 levels by western blotting or S phase entry by EdU incorporation. Results in panel C show mean ± SD, n = 3.

Prostacyclin (PGI_2_) is a potent anti-mitogen for VSMCs, and the anti-mitogenic effect of PGI_2_ is fully dependent on p27: PGI_2_ strongly increases p27 levels and blocks S phase entry in wild-type VSMCs yet fails to inhibit S phase entry in p27-null VSMCs [22]. We previously reported that cicaprost blocks the serum-dependent induction of Skp2 mRNA and protein and that forced Skp2 expression overcomes the anti-mitogenic effect of cicaprost in MEFs [24]. We observed similar effects of cicaprost on Skp2 in wild-type VSMCs (Fig. S3), and as expected these effects required the canonical PGI_2_ receptor, IP (Fig. S3). However, serum also induces miR221/222, and this induction is also strongly inhibited by cicaprost (Fig. 3A). Inhibition of serum-induced miR221/222 by cicaprost was seen in both WT and p27T187A VSMCs (Fig. S4), indicating that Skp2-mediated p27 degradation is not required for the effect. Importantly, forced expression of miR222 downregulated the levels of p27 (Fig. 3B) and promoted S phase entry (Fig. 3C) despite cicaprost treatment. Collectively, the results in Fig. S3 and 3 indicate that either the Skp2 or miR221/222 pathway is sufficient to *stimulate* p27 downregulation. However, since miR221/222 and Skp2 have compensatory effects on p27 (Fig. 2), both pathways must be inactivated to *prevent* p27 downregulation. Based on this reasoning, the fact that cicaprost inhibited both miR221/222 and Skp2 induction agreed well with its ability to block the downregulation of p27.

### Primary and Secondary Effects of PGI_2_ on miR221/222 and Skp2

**Figure 4:**
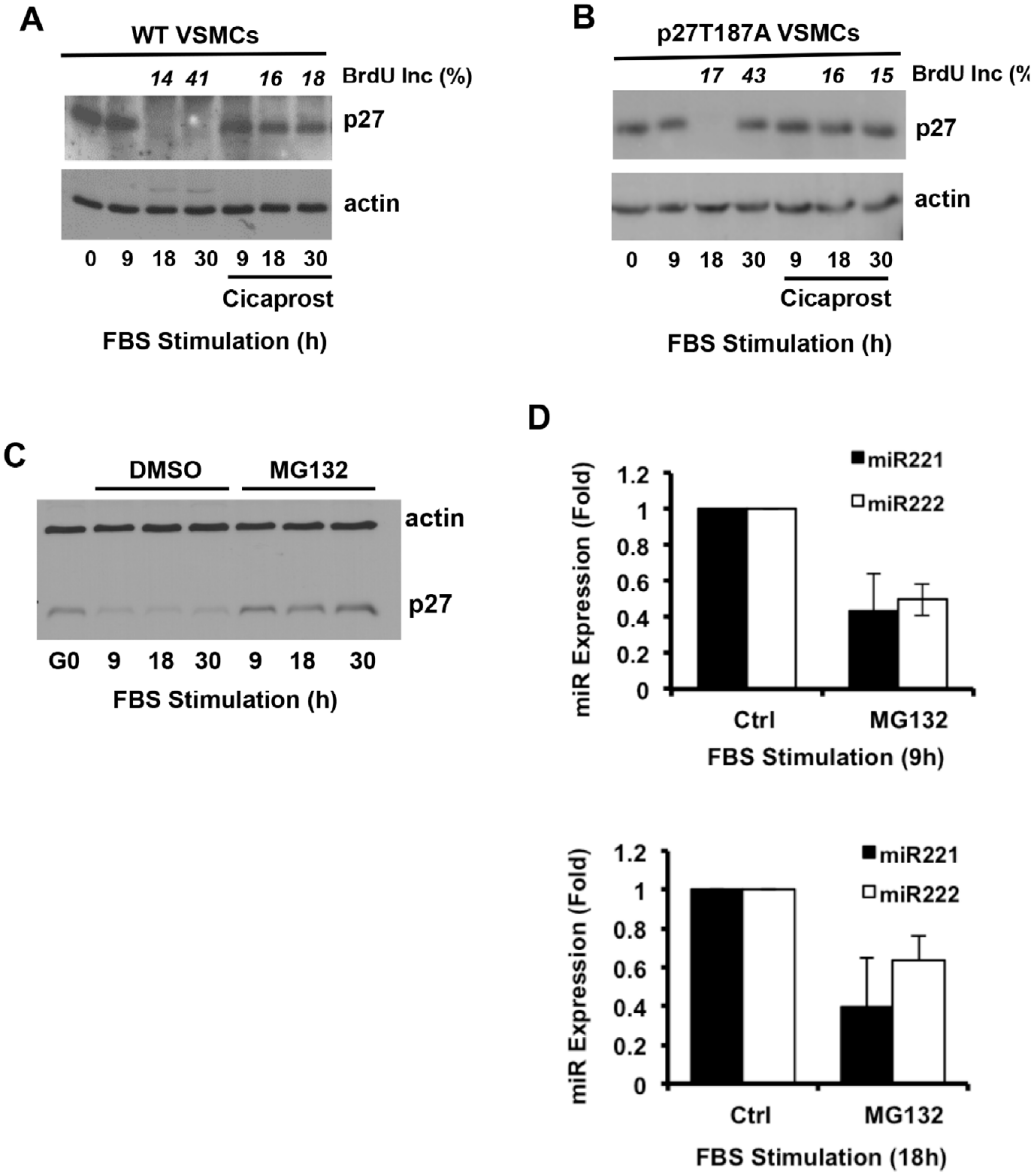
Regulation of G1 and S phase p27 by PGI_2_. (**A–B**) Quiescent early passage explant VSMCs from wild-type or p27T187A mice were stimulated with 10% FBS for 24 or 30 h in the absence or presence of 200 nM cicaprost. Total protein was extracted at the indicated times and analyzed by western blotting for p27 and actin (loading control). The percent EdU incorporation, determined from coverslips included in the experiments, is shown in italics. (**C–D**) Quiescent VSMCs were treated with 10 µM MG132 or DMSO (vehicle control) and stimulated with 10% FBS for the indicated times. Total protein was extracted and analyzed for p27 protein levels by western blotting and actin (loading control). Total RNA was extracted at 9 (n = 3) and 18 h (n = 2) and analyzed for miR221/222 by RT-qPCR. RT-qPCR results show mean ± SD. Note that G1 phase downregulation of p27 was evident 9 h after FBS stimulation in this experiment, affording us more time points to document the inhibitory effect of MG132 on G1 phase miR221/222.

As compared to wild-type VSMCs, the T187A mutation prevented serum-stimulated downregulation of p27 expression in S phase but not in G1 phase (Fig. 4A–B; compare 18 and 30 h). These results are similar to those reported initially by Malek et al. [7]. Yet cicaprost prevented the downregulation of p27 in G1 phase in both cell types (Fig. 4A–B). Thus, PGI_2_ can regulate p27 levels even when Skp2-mediated p27 proteolysis is absent. Others have reported that both G1 as well as S phase regulation of p27 are sensitive to the proteasome inhibitor, MG132 [7]. While this result would appear to conflict with a miR-dependent regulation of p27 in G1 phase, we unexpectedly found that MG132 inhibits G1 phase miR221/222 as well as p27 (Fig. 4C–D; 9 and 18 h). Thus, the effect of MG132 on G1 phase p27 downregulation may be indirect and involve miR221/222 (see Discussion).

**Figure 5:**
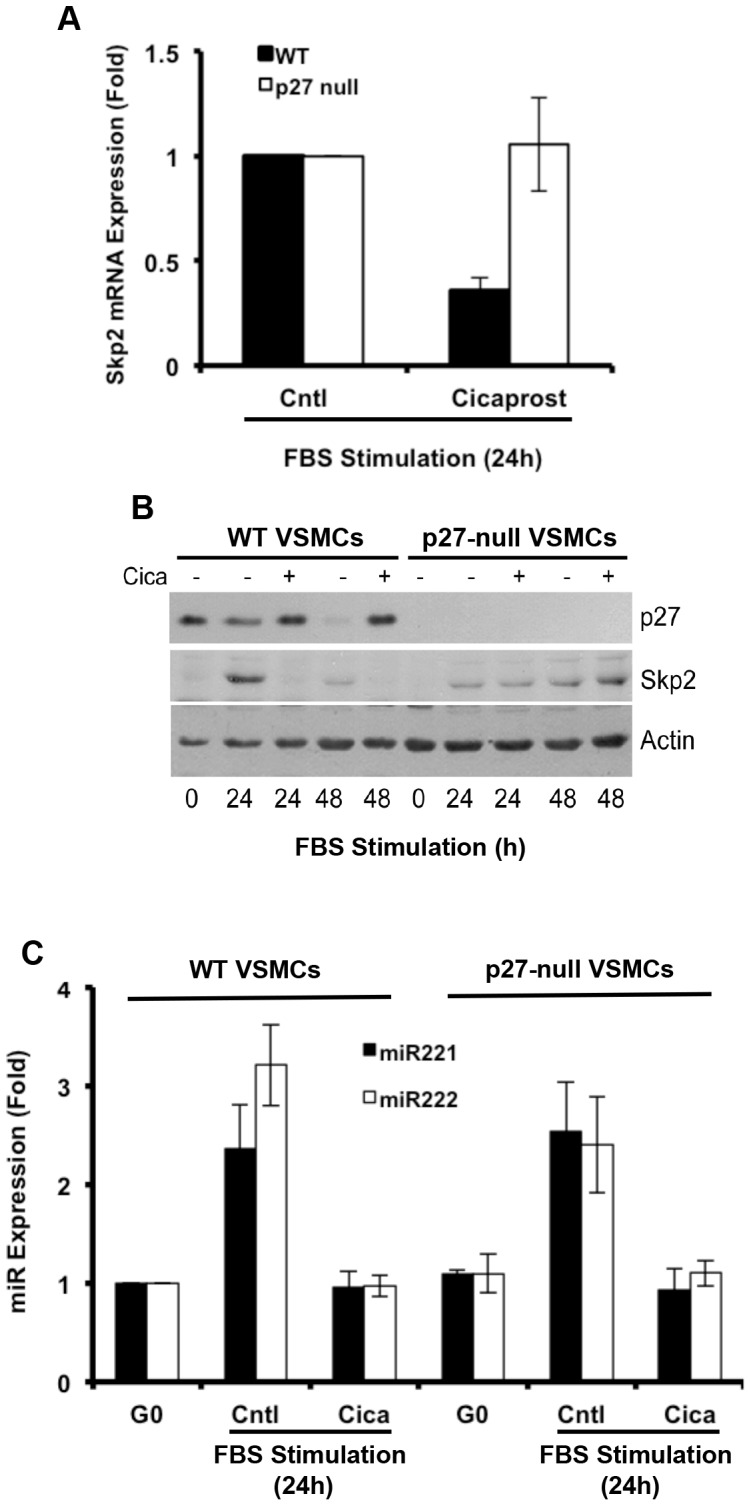
miR-221/222 is a primary target of PGI_2_. Quiescent early passage VSMCs from wild-type or p27-null mice were stimulated with 10% FBS in the absence (control; C) or presence of 200 nM cicaprost (cica). (**A**) Total RNA was extracted at 24 h, and Skp2 mRNA levels were determined by RT-qPCR. Results show mean ± SD, n = 2. (**B**) Total protein was extracted and analyzed by western blotting for p27, Skp2 and actin (loading control). **(C)** Total RNA was extracted at 24 h, and miR-221/222 levels were determined by RT-qPCR. Results show mean ± SD, n = 2.

Since miR221/222 and Skp2 must both be inhibited to prevent p27 down-regulation (refer to Fig. 2) and since Skp2 is required in S phase, we propose that miR221/222 has a major role tranducing the effect of cicaprost on G1 phase p27. This notion is consistent with our finding that the serum-dependent induction of miR221/222 precedes S phase entry (Fig. 2A; compare top and bottom panels). To explore the effects of PGI_2_ on miR221/222 and Skp2 in more detail, we compared the p27 response to cicaprost in VSMCs isolated from wild-type and p27-null mice. p27-null VSMCs are resistant to the anti-mitogenic effect of PGI_2_ [22]. Remarkably, we found that cicaprost was unable to regulate Skp2 mRNA or protein levels in p27-null VSMCs (Fig. 5A and B, respectively). Similarly, the mitogen-dependent induction of Cks1, a co-factor needed for efficient ubiquitin-mediated degradation of p27 [33], [34], was blocked by cicaprost in wild-type but not p27-null VSMCs (Fig. S5). Thus, the inhibitory effect of PGI_2_-IP signaling on Skp2 must be downstream of cell cycling rather than upstream. In striking contrast, the inhibitory effect of cicaprost on miR-221/222 persisted in p27-null VSMCs (Fig. 5C). We conclude that miR-221/222, rather than Skp2, is the primary target of PGI_2._ Nevertheless, PGI_2_ has an indirect effect on Skp2 and this contributes to the observed effect of PGI_2_ on overall p27 levels in VSMCs (see Discussion).

### Suppression of miR-221/222 Expression by cAMP

**Figure 6:**
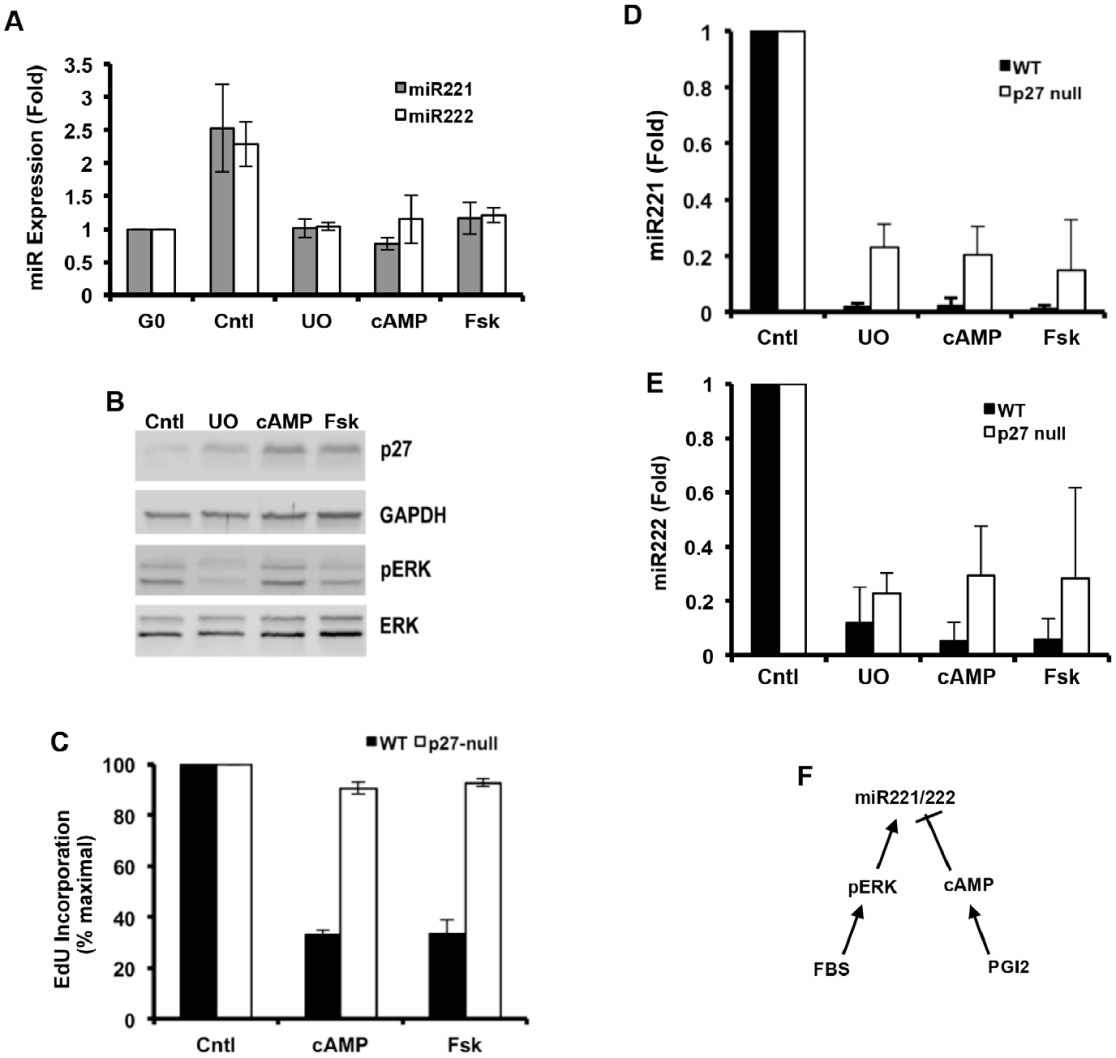
Effect of cAMP elevating agents on miR-221/222 and p27 expression. (**A–B**) Quiescent early passage VSMCs from wild-type were stimulated with 10% FBS in the absence (control) or presence of 50 µM U0126 (U0), 1 mM 8Br-cAMP, or 100 µM Forskolin (Fsk). In A, total RNA was extracted at 24 h, and miR-221/222 expression levels were determined by RT-qPCR. Results show mean ± SE, n = 3−4. In B, total protein was extracted at 24 h and analyzed by western blotting for p27, dually phosphorylated ERK (pERK), total ERK and GAPDH (loading control). (**C–E**) The experiment in A was repeated with wild-type and p27-null VSMCs in 6-well dishes containing coverslips and EdU. In C, coverslips were fixed at 48 h and stained for EdU; results are plotted relative to the FBS-treated control; n = 3. In D-E, total RNA was extracted at 24 h, and miR-221 or miR-222 expression levels were determined by RT-qPCR. Results show mean ± SD, n = 2. (**F**) miR-221/222 regulation by mitogens, ERK, PGI_2_, and cAMP.

We and others have shown that PGI_2_ increases intracellular cAMP in VSMCs; this effect requires the PGI_2_ receptor, IP, and the IP-dependent activation of Gs [35], [36]. Early studies showed that cAMP also upregulates p27 [37], but the responsible effector pathway has remained unknown. To determine if miR-221/222 might be the target of cAMP signaling, we treated VSMCs with 8-Br-cAMP or forskolin. Both treatments repressed miR-221/222 (Fig. 6A), increased p27 levels (Fig. 6B), and inhibited EdU incorporation (Fig. 6C). Importantly, the effect of cAMP on miR-221/222 is upstream of p27 and cell cycle progression because the inhibitory effect of 8-Br-cAMP and forskolin on miR-221/222 remained even in p27-null cells (Fig. 6D–E) where neither agent could prevent S phase entry (Fig. 6C). In contrast, Skp2 mRNA levels were down-regulated by cAMP in wild-type but not in p27-null VSMCs (Fig. S6), indicating that Skp2 regulation by cAMP is a secondary effect downstream of cell cycle arrest.

miR-221/222 induction is mediated by the ERK MAP kinase pathway [38], and in agreement with that study we found that the MEK/ERK inhibitor, U0126, reduced serum-stimulation of miR-221/222 in VSMCs (Fig. 6A). However, the inhibitory effect of 8-Br-cAMP and forskolin on miR-221/222 expression was not associated with reduced ERK activity (Fig. 6B). Thus, the cAMP effect on miR-221/222 reflects active inhibition that is independent of and antagonistic to ERK-dependent miR-221/222 induction (Fig. 6F). Although cAMP is canonically thought to signal through protein kinase A, it is now clear that cAMP also activates ion channels and the Epac family (guanine nucleotide exchange factors for Rap1 and Rap2) [39]. Elucidating the relative effects and redundancies of these multiple cAMP effectors on p27 regulation remains an important issue to resolve

## Discussion

### Regulation of p27 by miR-221/222 and Skp2

We show that miR221/222 redundantly regulate p27 levels post-transcriptionally, and this finding helps to explain why pronounced defects in p27 expression and cell cycling are not regularly observed in vitro or in vivo when Skp2-mediated p27 degradation is precluded by mutation of p27T187. Conversely, since either pathway is sufficient to downregulate p27, the inactivation of both pathways is required to efficiently block the decreased expression of p27 seen after mitogen stimulation. Our data also indicate that miR221/222 regulates p27 levels in G1 phase whereas Skp2 acts in S phase. Thus, while these two pathways can compensate for each other when one of these two pathways is inactive, they do appear to have sequential roles in regulating p27 during G1/S progression when both pathways are present and functional, at least in VSMCs.

Malek et al. [7] reported G1 phase regulation of p27 is sensitive to the proteasome inhibitor, MG132, and concluded that a proteasome-dependent mechanism regulated G1 phase p27 levels independently of T187 phosphorylation. At first glance, the microRNA-dependent regulation of p27 we described here would be inconsistent with this earlier work. However, we also show that MG132 effectively blunts miR221/222 induction. These results reveal an unanticipated connection between proteosomal degradation and miR221/222 expression. Whether this connection relates to proteasome-sensitive transcriptional regulation of the miR221/222 promoter or proteasome-dependent miR221/222 maturation/function remains to be determined. Others have reported that the KPC E3 ligase directly regulates G1 phase p27 degradation [9], but our ability to fully prevent serum-stimulated p27 downregulation by the combined inhibition of miR221/222 and the Skp2/T187 pathways indicates that KPC is either poorly expressed or minimally involved in p27 degradation in VSMCs.

Others have individually examined the roles of Skp2 or miR-221/222 on p27 and VSMC cycling. One of these studies reported reduced VSMC cycling after injury in Skp2-null mice [40], but this study does not speak to the importance of Skp2 in p27 regulation *per se* because Skp2 has p27-independent substrates effects [41], [42]. The same group concluded that Skp2 plays an important role in the anti-mitogenic effect of cAMP [43]. It is possible, however, that the changes in Skp2 expression reported by Wu et al. [8] are an indirect effect of miR-221/222-regulated p27 expression as described here. Still other studies describe a mitogen-stimulated induction of miR-221 [18] or both miR-221 and miR-222 [32] in VSMCs, and these effects inversely correlate with p27 levels. One of these studies [32] reported that transfection with an antisense oligonucleotide targeting miR-221/222 reduced neointima formation in injured arteries and reduced cycling in cultured VSMCs. We did not detect an inhibitory effect of miR-221/222 inhibition on VSMC cycling unless Skp2-mediated degradation was blocked.

In addition to p27, the miR-221/222 family downregulates expression of the closely related cdk inhibitor, p57^kip2^ [17], [32], [44]. However, our finding that proliferation of cultured p27-null VSMCs is resistant to cicaprost indicates that p57^kip2^ makes a relatively small contribution to the total cip/kip pool in cultured VSMCs. This notion is supported by the finding of increased atherosclerosis and vascular injury response to deletion of p27 alone ([19] and this report, respectively).

Skp2 is an E2F-regulated gene, and its expression is sensitive to alterations in the phosphorylation and activation state of Rb [29], [31]. Our previous studies show that PGI_2_ can inhibit cyclin E-cdk2 activity, Rb phosphorylation, and Skp2 gene expression [24], [35]. Based on these data and this report, we posit that PGI_2_ inhibits cell cycling by integrating miR221/222- and Skp2-dependent p27 expression. We suggest that the primary response to PGI_2_ is an inhibition of miR221/222 expression in G1 phase. Since Skp2 is not regulating G1 phase p27 in VSMCs, this effect should be sufficient to increase p27, which in turn could lead to decreased cyclin E-cdk2 activity, Rb phosphorylation, E2F release, and E2F-dependent Skp2 gene expression. A decrease in Skp2 expression could then account for increased expression of p27 in S phase [7], [9]. This model can explain why PGI_2_ upregulates steady-state p27 levels in both G1 and S phases and reduces Skp2 expression in wild-type VSMCs without directly targeting Skp2 itself.

## Materials and Methods

Animal work in this study was approved by the Institutional Animal Care and Use Committees of the Wistar Institute and University of Pennsylvania.

### In vivo Transcript Profiling

Four 6-mo male SMA-GFP mice were subjected to fine wire injury as described [45]. The uninjured femoral arteries and GFP-negative (dedifferentiated) regions of injured femoral arteries were isolated by microdissection and immediately placed in liquid nitrogen. Total RNA was isolated, and a 100 ng portion was subjected to linear amplification (Nugen WT ovation pico), and hybridized to Affymetrix mouse gene 1.0ST. Remaining RNA from the uninjured and injured tissues, respectively, was pooled and analyzed for differential microRNA expression by hybridization to Affymetrix miR-1.0 arrays.

Gene as well as miRNA expression microarray data were analyzed using Partek^®^ Genomics Suite^TM^ software (Partek Inc., St. Louis, MO). Raw signal intensities for mRNAs were subjected to background correction, quantile normalization, log_2_ transformation and probe-set summarization with the RMA (Robust Multichip Average) method. Since the data was paired, a 2-way-ANOVA test with factors condition (injured or uninjured) and Mouse ID was run to compute *p*-values of significance and an *F*-statistic for each probe-set. To correct for multiple testing, a two-class paired *t-test* was run within the SAM (Significance analysis of microarrays) software that computed a *q*-value, a measure of the false discovery rate (FDR) for each gene. The FDR values were integrated with the 2-way-ANOVA results. Genes that were significant at FDR cut-off of 5% and changed at least 1.7-fold in either direction in the injured group when compared to the control group were selected as the set of differentially expressed genes. The list of differentially expressed genes was subjected to GO enrichment analysis by running a Fisher Exact test against a background set to MoGene-v1.0_st microarray. From this analysis, genes were identified that belonged to the highly enriched GO categories, namely, Cell Division (GO: 51301), Cell Cycle Process (GO: 22402), Cell Proliferation (GO: 8283) and Cell Cycle (GO: 7049). This list of genes combined with list of changed miRNAs was used as input into Ingenuity Pathway Analysis software. For miRNA profiling, raw signal intensities were processed using RMA as described above. Following normalization, only mouse miRNA probe-sets (609) were retained for further analysis. A fold-change was computed for injury vs. control groups. Any miRNA that changed at least 1.7-fold in either direction was called differentially expressed.

Ingenuity Pathway Analysis software (Ingenuity^®^ Systems) was employed for exploration of interactions within and between mRNAs and miRNAs. Using the Build/Grow tool, a network of molecules, shown by experimental evidence in mouse, rat and human systems to act directly on p27 (CDKN1B), was generated. On this network, miRNAs that changed in response to injury and injury-regulated mRNAs belonging to the GO categories specified above, were overlapped and visualized.

### Cell Culture

Primary murine VSMCs were isolated from the aortae of male, 10–12-wk. wild-type C57BL/6 mice (Jackson Labs), p27^−/−^ mice on the C57BL/6 background mice (Jackson Labs), or p27T187A knock-in mice on the C57BL/6 background (kindly provided by Jim Roberts). The cells were isolated by explant culture as described [46], maintained in growth medium (1∶1 Dulbecco’s modified Eagle’s Medium (DME)/Ham’s F-12 supplemented with 2 mM L-glutamine and 10% FBS) and used between passage 2–5. For cell cycle experiments, 60–90% confluent monolayers of wild-type, p27^−/−^ or p27T187A VSMCs were grown in 35-mm (RT-qPCR and S phase assays) or 100-mm culture dishes for western blotting experiments. The cells were G0 synchronized by incubation in serum-free DME containing 1 mg/ml heat-inactivated fatty acid-free BSA (DME-BSA) for 48 h before stimulation with fresh growth medium in the absence or presence of 200 nM cicaprost (kindly provided by Bayer Schering). In some experiments, VSMCs were serum starved for 48 h and then pre-incubated in fresh serum-free medium 10 µM MG132 (Sigma; #474790) for 20 min. FBS was then added to a final concentration of 10%, and the cells were stimulated for the indicated times.

To monitor S phase entry, VSMCs were plated in wells containing coverslips and EdU (Invitrogen) at a final concentration of 10 µM. The cells were fixed, permeablized cells and analyzed using the Click-iT EdU imaging kit (Invitrogen). Nuclei were identified by Dapi-staining. Images were captured using a Nikon Eclipse 80i microscope, 20X/0.45 PL Plan Fluor objective, Hamamatsu C4742-95 digital camera and camera controller. Images were captured using Image-Pro Plus software, and the number of EdU-positive and Dapi-positive nuclei was manually counted. In some experiments, S phase entry was determined by BrdU incorporation as described [24], [35].

### miRNA Knock-down and Ectopic Expression

Mouse VSMCs were transfected in serum-free conditions with control (Applied Biosystems; AM17010) or anti-miR-222 (Applied Biosystems; AM11376) oligonucleotides at a final concentration of 100 nM using Lipofectamine 2000 (Invitrogen) in 100-mm dishes. After 4 h, the medium was replaced with OPTI-MEM overnight. The medium was then replaced with DME/BSA and incubated for a total of 48 h from the beginning of transfection. Cells were then stimulated directly in the dish with regular growth medium for 24 h. For rescue experiments, we transfected near confluent VMSCs in 100-mm dishes with 5 µg of either an expression plasmid for microRNA-222 (Origene; SC400931) or pCDNA (control) using 40 µl Lipofectamine 2000. After 4 h, the transfected cells were allowed to recover overnight in regular growth medium. The cells were then starved for 48 h in DMEM/BSA and directly stimulated with fresh growth medium. VSMCs were infected with adenoviruses encoding LacZ and human Skp2 largely as described [29], [31].

### Femoral Artery Injury and Analysis

Fine-wire femoral artery injury was performed as described [45] on 5–7 mo wild-type, p27T187A knock in, and p27-null male mice on the C57BL/6 background as described. Arteries were perfusion fixed in Prefer (Anatech Ltd.), embedded in paraffin, and 5-micron sections were stained using Accustain Elastic Stain (Sigma-Aldrich) to identify the internal and external elastic laminae and peak injury sections. Luminal, medial, and neointimal areas from three peak injury sections were quantified using Image Pro software, and the mean values were used to calculate neointimal/medial ratios and percent luminal stenosis for each mouse. Cell proliferation was assessed by anti-Ki67 (DAKO; M7249) staining of deparaffinized injury sections that had undergone antigen retrieval in 10 mM sodium citrate, pH 6 for 20 min with heat. Images were captured with Image Pro software at 20× magnification as described below. The number of Ki67-positive nuclei per peak injury section was counted manually.

### Immunoblotting

Mouse VSMCs were collected and lysed as described [47]. Total protein concentration was determined by Coomassie binding (Bio-Rad, Hercules, CA). Equal amounts of protein (15–25 µg) were resolved on reducing SDS mini-gels and immunoblotted using antibodies specific for p27 (BD Biosciences Pharmingen), Skp2 (Zymed Laboratories), dually phosphorylated ERK (Cell Signaling Technology), ERK1/2 (Cell Signaling Technology), GAPDH (Santa Cruz Biotechnology; loading control), or actin (Santa Cruz Biotechnology; loading control). The resolved proteins were detected using ECL (Amersham).

### Quantitative Real-Time Reverse Transcriptase-PCR (RT-qPCR)

Total RNA was extracted from collected cell pellets using 1 ml TRIZOL reagent (Invitrogen) followed by reverse transcription using ∼100 ng of total RNA as described previously [48]. An aliquot (20%) of the cDNA was subjected to RT-QPCR using TaqMan universal PCR Master Mix (Applied Biosystems). RT-qPCR for Skp2 mRNA and 18S rRNA were performed as described previously [48]. For mouse Cks1, RT-QPCR was performed using SYBR Green PCR Master Mix (Applied Biosystems), 0.5 µm forward primer (5′-GCAATCATGTCCCACAAACA-3′) and 0.5 µm reverse primer (5′-GACCAGCTTGGCTATGTCCT-3′. For the analysis of miR-221 and miR-222 expression, total RNA was isolated from cells or isolated aortae with TRIZOL and reverse transcribed using ∼50 ng of RNA in a 15-µl reaction with TaqMan microRNA reverse transcription kit (Applied Biosystems). An aliquot (20%) of the reaction was used for RT-qPCR using TaqMan universal master mix, Mature microRNA assay ID #524 and #2276 (Applied Biosystems), and snoRNA202 (Assay ID #1232, Applied Biosystems). RT-qPCR results were calculated using the standard curve or ddCt methods using 18S rRNA or snoRNA202 as the reference for mRNAs and miRNAs, respectively.

## Supporting Information

Figure S1 **Skp2-mediated p27 degradation is not required for the in vivo response to vascular injury.** The left femoral arteries of male mice (5–6 months old) were subjected to fine-wire induced femoral artery injury. The contralateral artery of each mouse was mock injured and used as control. Mice were sacrificed 14 days after the injury procedure. **(A)** Cross sections of uninjured and injured femoral arteries stained for elastin. **(B)** Luminal stenosis of injured wild-type (n = 13), p27-null (n = 14), and p27T187A (n = 10) mice graphed as box and whisker plots where whiskers show minimum and maximum values. **(C)** Representative images of Ki67-labeled nuclei in peak injury sections. **(D)** Ki67 results from wild-type (n = 8), p27-null (n = 8), and p27T187A (n = 8) mice graphed as box and whisker plots. *p* values in C and E were calculated using a 2-tailed Mann-Whitney test.
(TIF)

Figure S2 **Effect of anti-miR222 on miR221 and miR222.** Early passage VSMCs from wild-type mice were transfected with control anti-miR (Cntl) or anti-miR222, serum-starved (G0) and then stimulated with 10% FBS for 24 h. Cells were collected, lysed and analyzed by RT-qPCR for miRNA221 and miR222. Results show mean ± SD, n = 2.
(TIF)

Figure S3 **Cicaprost inhibits Skp2 mRNA and protein expression while Skp2 overexpression rescues S phase entry.** Serum-starved VSMCs from wild-type and IP-null mice were incubated with 10% FBS in the absence (control; C) or presence of 200 nM cicaprost (Cica) for 24 h. (**A)** RNA was isolated and analyzed by RT-qPCR for Skp2 mRNA. Results show mean ± SD, n = 2. **(B)** Total protein was analyzed by western blotting for Skp2, cyclin A and cdk4 (loading control). **(C)** Wild-type VSMCs were infected with adenoviruses encoding LacZ or human Skp2, serum-starved, and then incubated with 10% FBS and BrdU in the absence (control; C) or presence of 200 nM cicaprost (Cica). BrdU Incorporation was determined by immunofluorescence microscopy. Results show mean ± SD, n = 2.
(TIF)

Figure S4 **PGI2 inhibits miR221/222 expression in wild-type and IP-null VSMCs.** Quiescent early passage VSMCs from wild-type and p27T187A mice were stimulated with 10% FBS in the absence (control) or presence of 200 nM cicaprost for 24 h. Cells were analyzed by RTqPCR for miR221 and miR222. Results show mean ± SE, n = 4 for WT and n = 3 for p27T187A.
(TIF)

Figure S5 **PGI2 inhibits expression of Cks1 in VSMCs.** Quiescent early passage VSMCs from wild-type mice were stimulated with 10% FBS in the absence (control) or presence of 200 nM cicaprost for the indicated times. (A) Cells were analyzed by RT-qPCR for Cks1 mRNA. Results show mean ± SD, n = 2. (B) Lysates were immunoblotted for Cks1 and actin (loading control).
(TIF)

Figure S6 **The inhibitory effect of cAMP on Skp2 mRNA is downstream of cell cycle arrest.** The experiment in Fig. 6B-C was analyzed for Skp2 mRNA. Results show mean ± SD, n = 2.
(TIF)

Table S1 **Transcript profiling results of injured vs. uninjured mouse femoral arteries.** Results show mRNAs in GO category “cell cycle.”
(XLSX)

Table S2 **Transcript profiling results of differentially expressed miRNAs in injured vs. uninjured mouse femoral arteries.**
(XLSX)
