# Supplementary figures and images for: miR-221/222 Compensates for Skp2-Mediated p27 Degradation and Is a Primary Target of Cell Cycle Regulation by Prostacyclin and cAMP
Source: PLoS One. 2013 Feb 7;8(2):e56140. doi: 10.1371/journal.pone.0056140 (PMC3567044; doi:10.1371/journal.pone.0056140)

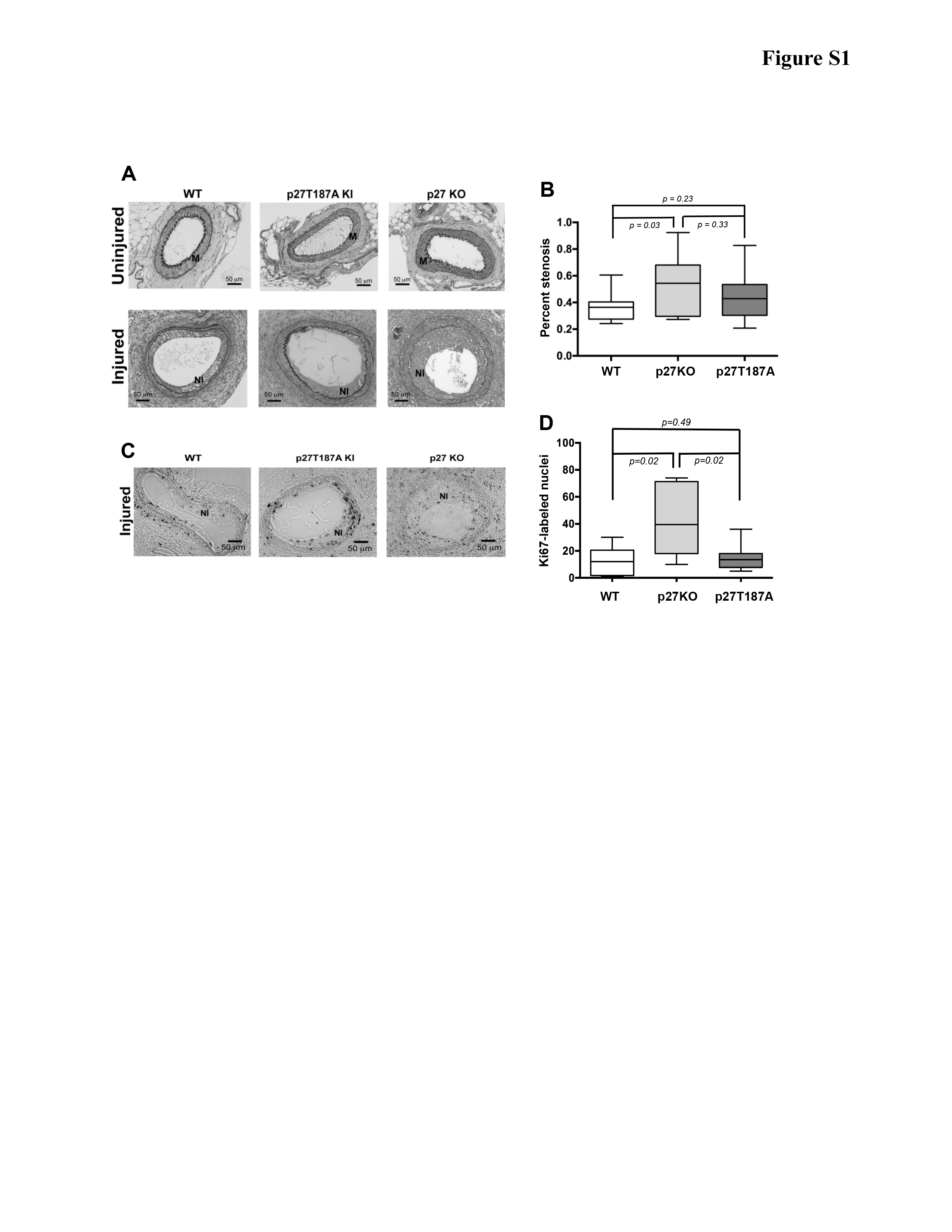

Supplement: Figure S1 — Skp2-mediated p27 degradation is not required for the in vivo response to vascular injury. The left femoral arteries of male mice (5–6 months old) were subjected to fine-wire induced femoral artery injury. The contralateral artery of each mouse was mock injured and used as control. Mice were sacrificed 14 days after the injury procedure. (A) Cross sections of uninjured and injured femoral arteries stained for elastin. (B) Luminal stenosis of injured wild-type (n = 13), p27-null (n = 14), and p27T187A (n = 10) mice graphed as box and whisker plots where whiskers show minimum and maximum values. (C) Representative images of Ki67-labeled nuclei in peak injury sections. (D) Ki67 results from wild-type (n = 8), p27-null (n = 8), and p27T187A (n = 8) mice graphed as box and whisker plots. p values in C and E were calculated using a 2-tailed Mann-Whitney test. (TIF) [file pone.0056140.s001.tif]

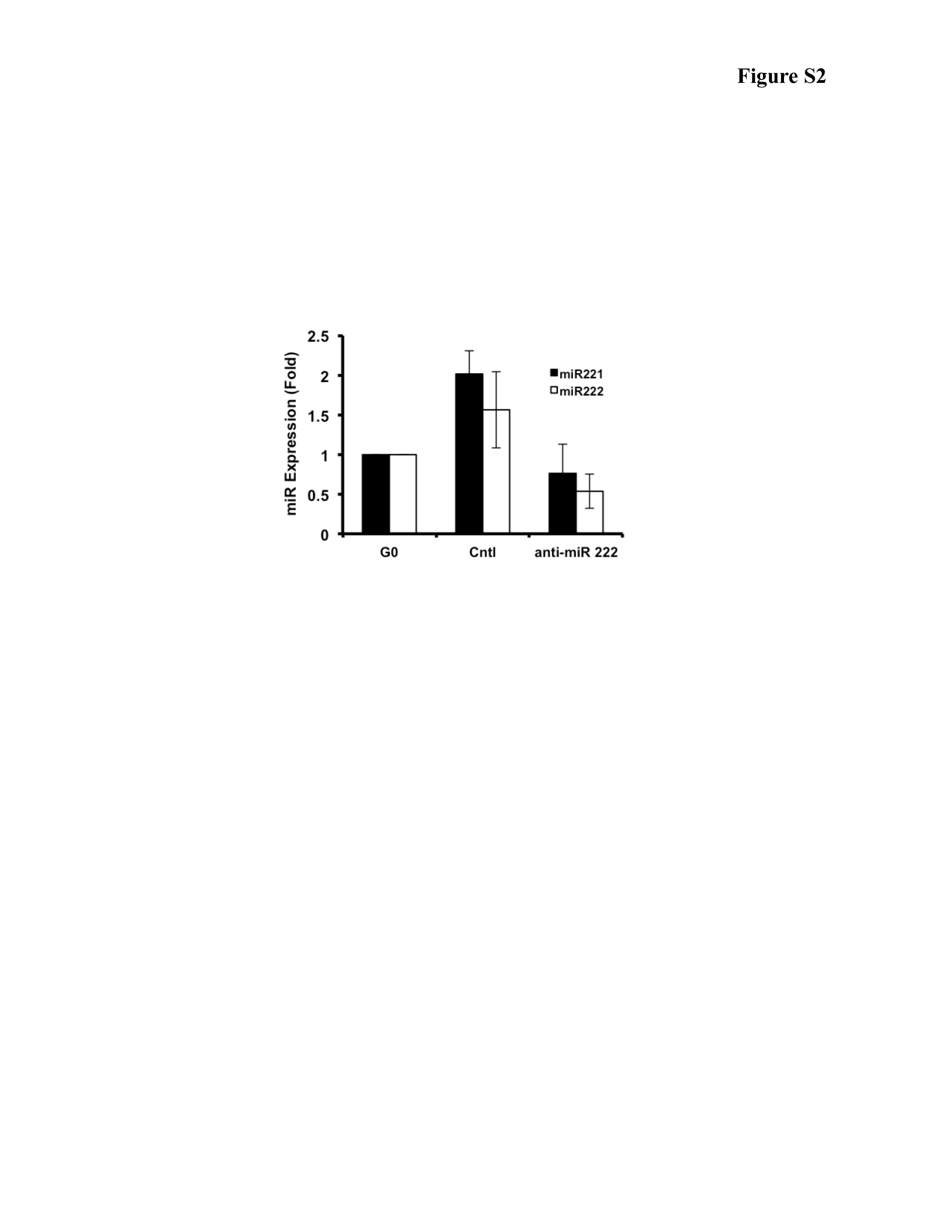

Supplement: Figure S2 — Effect of anti-miR222 on miR221 and miR222. Early passage VSMCs from wild-type mice were transfected with control anti-miR (Cntl) or anti-miR222, serum-starved (G0) and then stimulated with 10% FBS for 24 h. Cells were collected, lysed and analyzed by RT-qPCR for miRNA221 and miR222. Results show mean ± SD, n = 2. (TIF) [file pone.0056140.s002.tif]

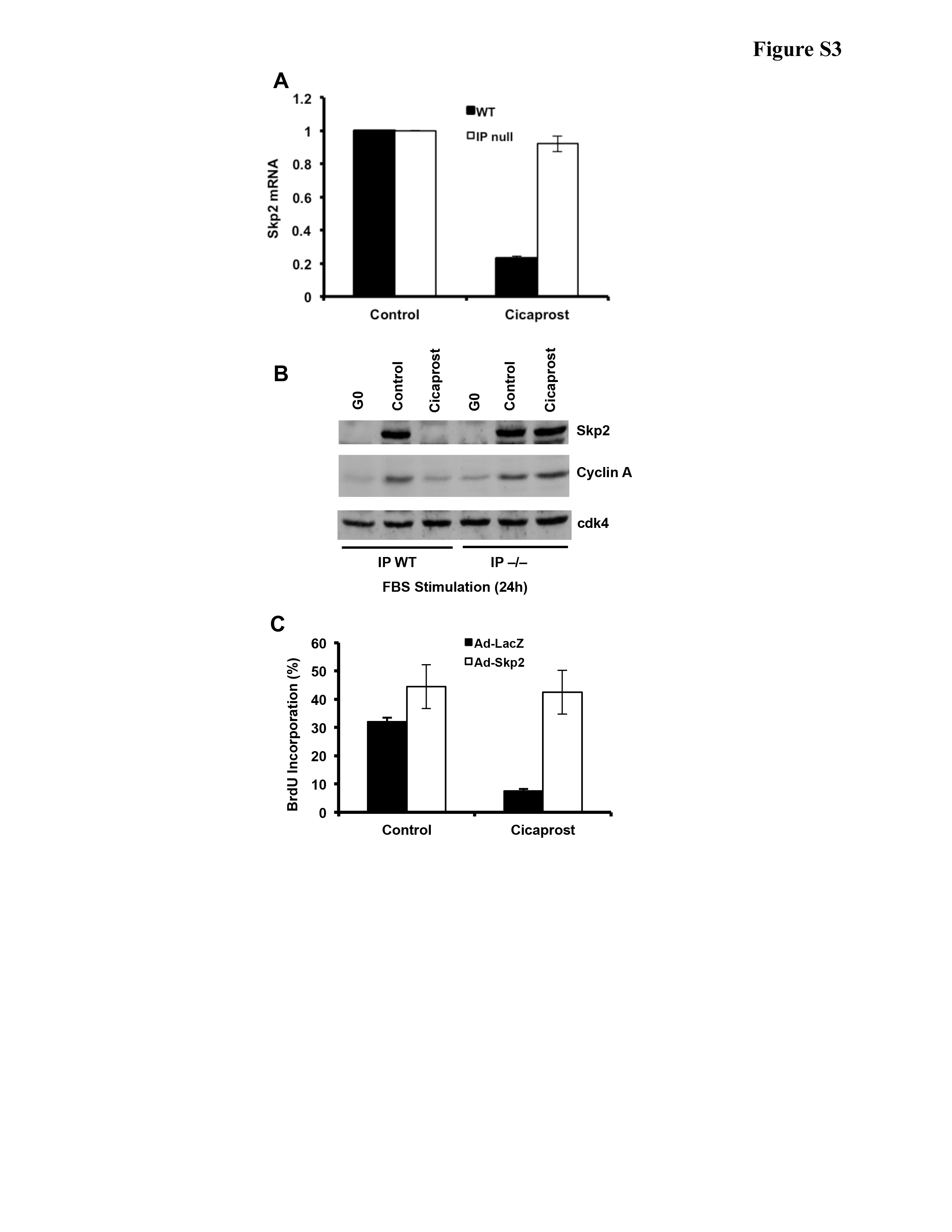

Supplement: Figure S3 — Cicaprost inhibits Skp2 mRNA and protein expression while Skp2 overexpression rescues S phase entry. Serum-starved VSMCs from wild-type and IP-null mice were incubated with 10% FBS in the absence (control; C) or presence of 200 nM cicaprost (Cica) for 24 h. (A) RNA was isolated and analyzed by RT-qPCR for Skp2 mRNA. Results show mean ± SD, n = 2. (B) Total protein was analyzed by western blotting for Skp2, cyclin A and cdk4 (loading control). (C) Wild-type VSMCs were infected with adenoviruses encoding LacZ or human Skp2, serum-starved, and then incubated with 10% FBS and BrdU in the absence (control; C) or presence of 200 nM cicaprost (Cica). BrdU Incorporation was determined by immunofluorescence microscopy. Results show mean ± SD, n = 2. (TIF) [file pone.0056140.s003.tif]

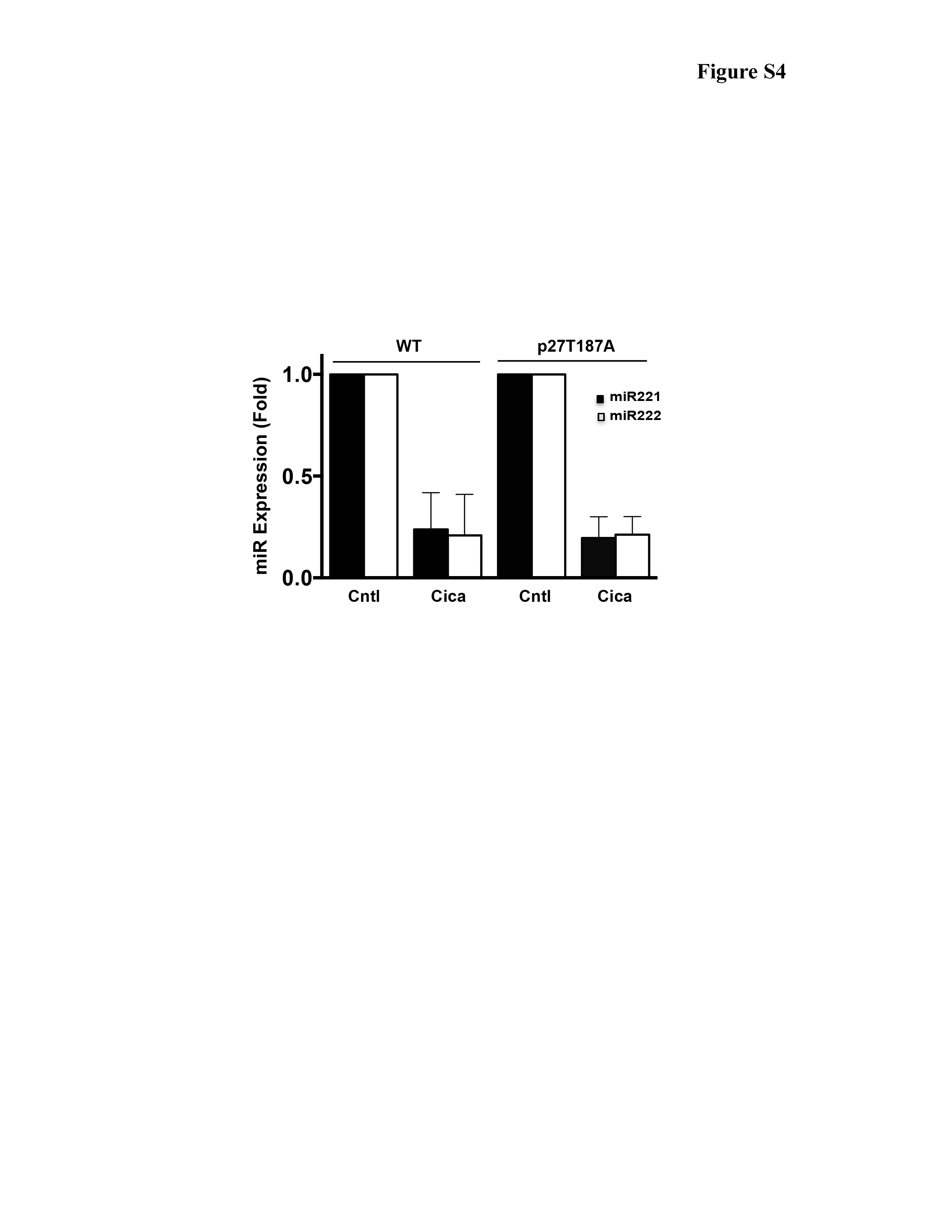

Supplement: Figure S4 — PGI2 inhibits miR221/222 expression in wild-type and IP-null VSMCs. Quiescent early passage VSMCs from wild-type and p27T187A mice were stimulated with 10% FBS in the absence (control) or presence of 200 nM cicaprost for 24 h. Cells were analyzed by RTqPCR for miR221 and miR222. Results show mean ± SE, n = 4 for WT and n = 3 for p27T187A. (TIF) [file pone.0056140.s004.tif]

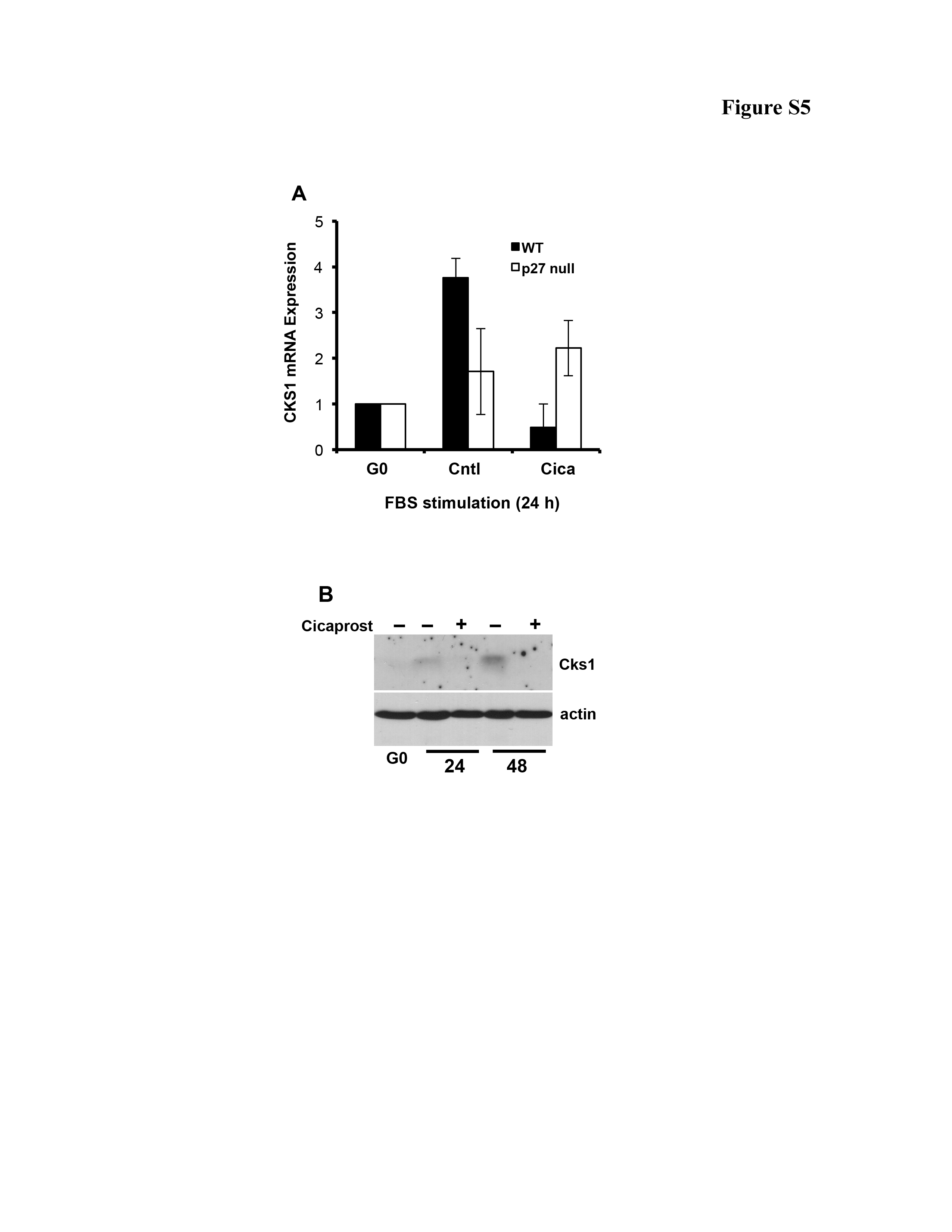

Supplement: Figure S5 — PGI2 inhibits expression of Cks1 in VSMCs. Quiescent early passage VSMCs from wild-type mice were stimulated with 10% FBS in the absence (control) or presence of 200 nM cicaprost for the indicated times. (A) Cells were analyzed by RT-qPCR for Cks1 mRNA. Results show mean ± SD, n = 2. (B) Lysates were immunoblotted for Cks1 and actin (loading control). (TIF) [file pone.0056140.s005.tif]

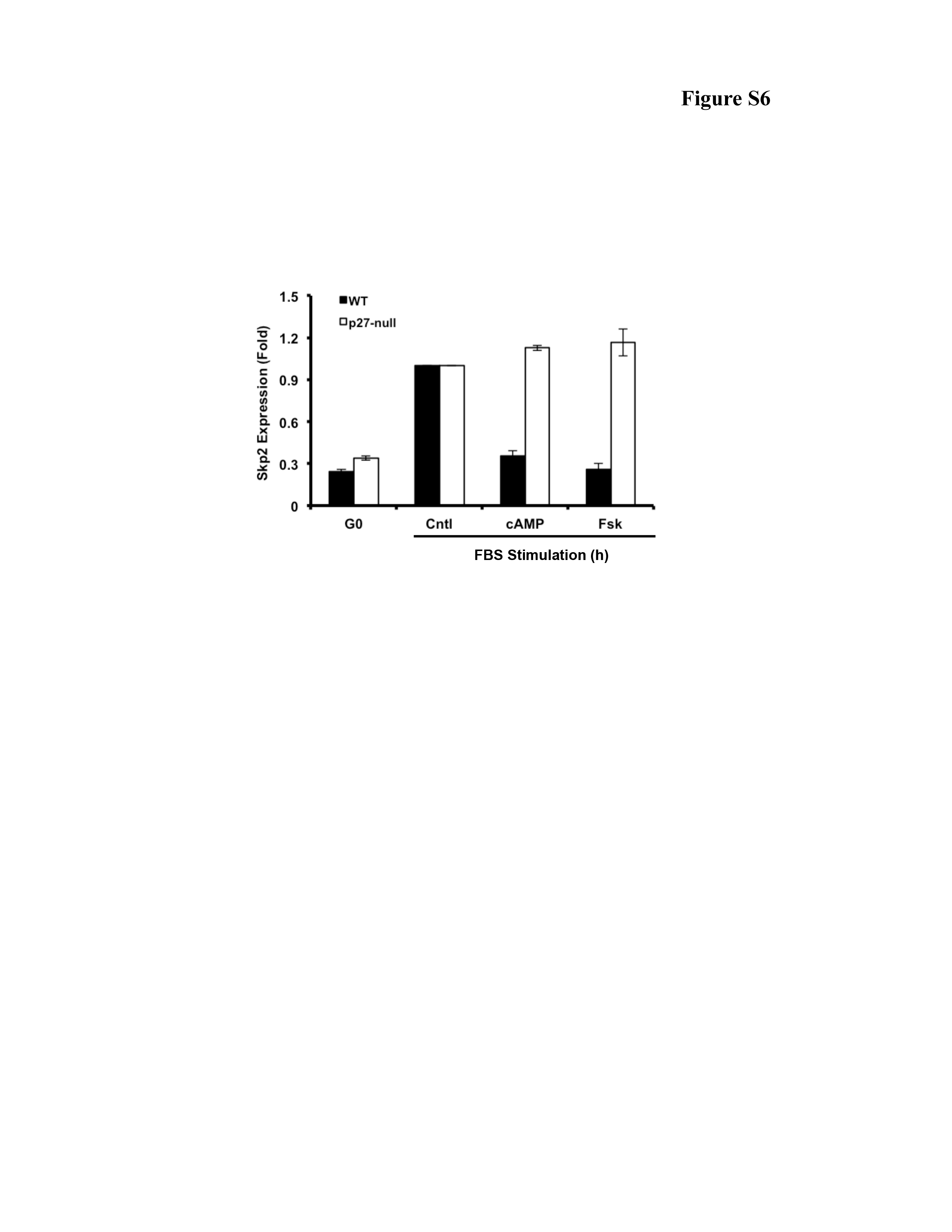

Supplement: Figure S6 — The inhibitory effect of cAMP on Skp2 mRNA is downstream of cell cycle arrest. The experiment in Fig. 6B-C was analyzed for Skp2 mRNA. Results show mean ± SD, n = 2. (TIF) [file pone.0056140.s006.tif]
